# Excessive teleological thinking is driven by aberrant associations and not by failure of reasoning

**DOI:** 10.1016/j.isci.2023.107643

**Published:** 2023-08-15

**Authors:** Joan Danielle K. Ongchoco, Santiago Castiello, Philip R. Corlett

**Affiliations:** 1 Yale University, New Haven, CT 06520, USA

**Keywords:** Health sciences, Human activity in medical context, Association analysis

## Abstract

Teleological thought — the tendency to ascribe purpose to objects and events — is useful in some cases (encouraging explanation-seeking), but harmful in others (fueling delusions and conspiracy theories). What drives excessive and maladaptive teleological thinking? In causal learning, there is a fundamental distinction between associative learning versus learning via propositional mechanisms. Here, we propose that directly contrasting the contributions of these two pathways can elucidate the roots of excess teleology. We modified a causal learning task such that we could encourage associative versus propositional mechanisms in different instances. Across three experiments (total N = 600), teleological tendencies were correlated with delusion-like ideas and uniquely explained by aberrant *associative* learning, but not by learning via *propositional* rules. Computational modeling suggested that the relationship between associative learning and teleological thinking can be explained by excessive prediction errors that imbue random events with more significance — providing a new understanding for how humans make meaning of lived events.

## Highlights

• People spuriously believe that events happen for a reason, but we do not know why
• Kamin blocking can reveal the causal learning roots of excessive teleological thought
• We need to distinguish between blocking via associations vs. propositional reasoning
• Spurious teleological thinking correlates with associative, not propositional blocking

## Graphical abstract

**Figure:**
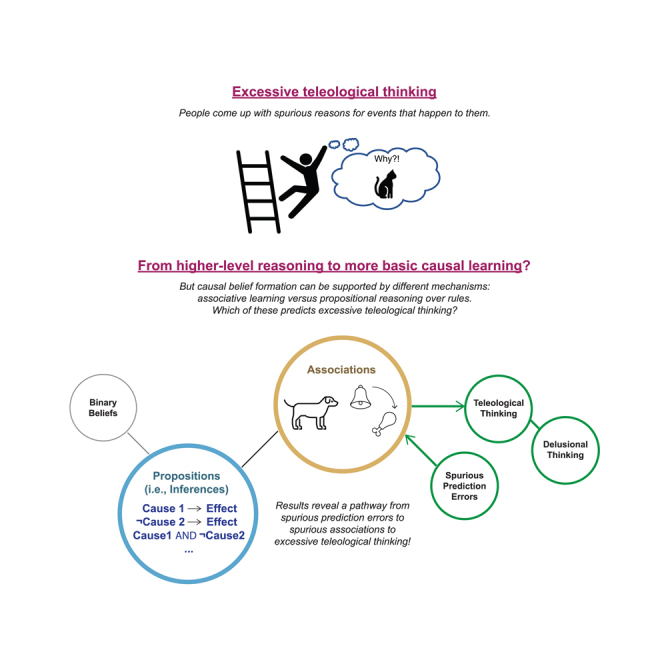

## Introduction

One of the most fundamental aspects of human cognition is our tendency to inquire about the purpose of objects. Early in development, children might encounter an object (e.g., the tail of their dog), and ask “what is this for?”.^1^ This also applies to events that unfold around us. People often ascribe purpose to random events. For instance, when people are given an event (e.g., “a power outage happens during a thunderstorm and you have to do a big job by hand”), and an outcome (e.g., “you get a raise”), they might attribute the pay raise to the power outage, even if this event does not have a direct causal relationship to the outcome.^2^ These types of explanations — where purpose is attributed to random or unexpected events — constitute *excess teleological thinking*. This phenomenon appears to occur more for unexpected events, or events that are out of our control.^3^ Animals that behave unpredictably are attributed more human characteristics and agency,^4^ and people ascribe more causal power to life events that are unexpected.^5^ Teleological thinking can help us find meaning in misfortune.^3^ At its extremes though, teleological thinking can fuel conspiracy beliefs and delusional thought.^6^^,^^7^

Ascribing purpose to objects and events around us has been typically explored in the context of higher-level cognition. Teleological thinking seems to be informed in part by cultural upbringing,^8^ but also by potentially a more domain-general bias to look out for design and intentionality to understand our social and natural worlds.^9^^,^^10^ But while it might be intuitive to think of teleological thinking as part of our reasoning capacities (and indeed, scientific understanding has been shown to co-occur with proclivities toward teleological explanations^11^), the *over*-ascription of purpose has been shown to actually reflect *less* conscious and careful thinking, such that people who engage in less cognitive reflection (giving fewer intuitive but wrong answers to cognitive puzzles) show greater tendencies toward teleological thought.^12^ One possibility is that more analytical thinking and reflection leaves less room for teleology. Alternatively, there may be a separate pathway to excess teleology: other low-level, and largely automatic processes might enable and deepen teleological thought.

Here, we propose that excess teleological thinking, the tendency to ascribe inappropriate purpose to objects and events, may be rooted not just in biases, but also in how we perceive and learn about causal relationships in the world.

In the animal literature, the mechanisms of causal learning have been well-explored. The capacity to prioritize what associations to learn versus which are redundant is critical. Blocking is a canonical behavioral phenomenon that emphasizes this point.^13^^,^^14^ It was first reported in 1969 by Leon Kamin in a fear conditioning procedure: in rats, prior learning that a tone predicts an electric shock blocks new learning about any causal power of a light (presented in conjunction with the tone). This observation led to an emphasis on prediction error in learning — mere contiguity is not enough. Instead, learning proceeds to the extent that outcomes are surprising.^15^ No surprise, no learning. This phenomenon of blocking has been demonstrated in monkeys,^16^ mice,^17^ pigeons,^18^ and crickets,^19^ and has been applied to human causal learning — humans form beliefs about causal^20^^,^^21^ and social relationships^22^ that are subject to blocking, implying that perhaps conditioning and believing may share a common underlying apparatus.^20^^,^^23^^,^^24^

Recent work has also suggested, however, that blocking in causal learning does not rely merely on these low-level predictive mechanisms, but also on more explicit reasoning over rules or “propositions”.^25^^,^^26^ When outcomes are no longer binary (allergy vs. no allergy), but more continuous (no allergy, allergy, strong allergy), there is room for more complex interactions between cues beyond simple competition. For example, suppose people are trained with an ‘additivity’ rule that two foods that cause an allergy might add together to cause a strong allergy. If pre-trained on this additivity mechanism, people may not only ignore the blocked cue as irrelevant, they may deduce that it does not cause the allergy — since, if it did, the combined effect of the blocking and blocked cue ought to have been a strong allergy. Some have suggested that rodents, and perhaps even invertebrates, are capable of such learning over propositions.^27^

This distinction in causal learning via *associations* versus via *propositional reasoning* comes into the fore in other areas of cognitive science, from debates on the nature of mental representations,^28^^,^^29^^,^^30^ to efforts to build networks that can effectively explain and predict mental phenomena.^31^^,^^32^ Here, we want to address the role that this distinction may play in teleological thinking head-on. Despite teleological thinking having been studied primarily as a reasoning phenomenon, it may be that teleological thinking is underwritten by a more basic causal learning mechanism. Dissociating between aberrant associations (via causal learning) and aberrant reasoning may elucidate how and why teleological thinking can go from helpful to maladaptive.

In the present studies, we asked whether excessive teleological thinking would at all be related to this basic causal learning mechanism in the first place. For teleological thinking, we used a standard validated measure from the literature, the *Belief in the Purpose of Random Events* survey.^2^ In this survey, people are given two different (and unrelated) events, and are asked to what extent one event could have “had a purpose” for the other (e.g., “power outage happens during a thunderstorm and you have to do a big job by hand” and “you get a raise”). Such a survey has been tested and correlated with a battery of different measures now, and remains the only task (to our knowledge) investigating teleological thinking in the context of *events* that occur in one’s life, and not just in the context of ascribing purpose to objects.

**Figure 1:**
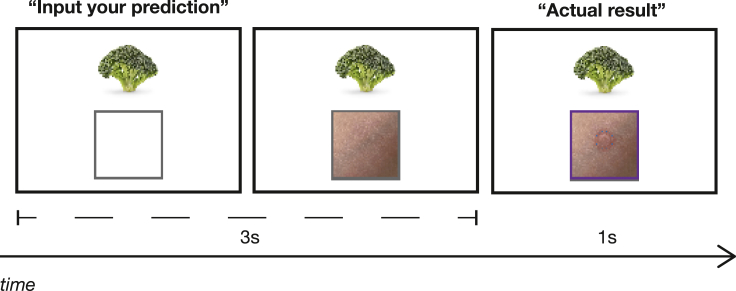
A caricature of a single trial In the first panel, subjects are first presented with a food cue. In the second panel, their prediction is visually depicted in the response box. In the third panel, subjects received feedback of what the actual outcome was.

For causal learning, we used *blocking* as our main measure of interest, which we could setup using the Kamin blocking paradigm.^13^ Participants were shown a food cue and asked to predict an allergic reaction (see Figure 1). The cue-outcome contingencies are depicted in Table 1, and the learning, blocking, and test phases were adapted from previous work.^23^^,^^33^ To the extent that blocking reflects the ability to prioritize relevant information from redundant ones, blocking *failures* would reflect a tendency to learn more from irrelevant cues and overpredict causal relationships between items that should not be associated. Such a tendency may underwrite excessive teleological thinking.

**Table 1:** Trial types for the causal learning paradigms

Pre-Learning	Learning	Blocking	Test
I+(+)^a^	A1+^b^	A1B1+	B1
J+(++)	A2+	A2B2+	B2
IJ+(+++)	C1-	C1D1+	D1
	C2-	C2D2+	D2
		UV-	Z
		WX-c^,^^d^	
		YZ-	

a The correct responses are indicated as −/+/++/+++ symbols (in parenthesis we present the correct responses for the Additive case).

b To increase task difficulty, subjects learned about the causal relationships between four cues (two types of A cues, and two types of C cues — and thus, there were two potential “blocked” (B) cues as well, etc).

c To balance out responses (allergy vs. no allergy), we added more no-allergy controls in the form of WX- and YZ-.

d Experiment 1 did not include the trial types UV- nor WX-. We add them for Experiment 2 and 3 to increase difficulty.

More importantly though, with the Kamin blocking paradigm, we can test two different causal learning pathways, and directly compare their predictive power to see which may explain more variance in teleological thinking — in a way that might reconcile existing tensions in how we understand teleological thinking. The classic non-additive blocking paradigm^13^^,^^33^ would speak to causal learning via associations (i.e., prediction error), while the additive blocking paradigm^25^^,^^27^ would speak to causal learning via more explicitly learned rules (i.e., propositional reasoning). The critical manipulation that separates non-additive from additive blocking is what happens in a *pre-learning* phase — in which subjects either learn an additivity rule or not, which has been suggested to move people to complete the blocking task through explicit reasoning, and not just learning.

Ultimately, additive and non-additive blocking might be related to each other, but additive blocking may constitute additional machinery on top of basic blocking, such that learning explicit propositions increases the magnitude of blocking. By distinguishing between the two, we can know whether excessive teleological thinking is a failure of one over the other.

(1) If excessive teleological thinking reflects a failure of reasoning, as implied in previous work,^11^^,^^12^ then we should see teleological thinking correlate with additive blocking (via reasoning over propositions and rules). In other words, excessive teleological thinking could be intimately related to how people *infer* causal relationships based on what they know of the rules they’ve been taught about the world.
(2) If excessive teleological thinking comes down to aberrant associations, then teleological thinking should correlate with non-additive blocking (via learned associations and aberrant prediction errors). In other words, excessive teleological thinking may be intimately related to how people attend to and prioritize different encountered cues in the world.

Moreover, the direction of this relationship can also matter.

(1) According to the cognitive reflection data,^12^ there might be a negative relationship between teleology and additive blocking, such that stronger propositional reasoning abilities leads to less teleological thinking.
(2) Given the relationship between scientific understanding and teleology,^11^ it is possible that stronger propositional reasoning abilities might correlate with excessive teleological thinking.

In the three experiments here, we followed a continuum approach — exploring the relationship between teleological thinking and causal learning in the general population. Alongside these two key variables, we also collected information on people’s tendencies (1) to subscribe to delusion-like beliefs (via the Peters’ Delusions Inventory^34^ and the Revised Greene Paranoid Thoughts Scale^35^), (2) to engage in all-or-none (or binary) thinking (via a task that tracks the extent to which participants binarize their beliefs — such that beyond a certain threshold of credence, people will lean toward full credulity, and below, they will deny any credence^36^; see Table 2; this is especially critical for delusional thinking, since previous work suggests that people with delusion-like beliefs seem to engage in ‘all or none’ belief updating, jumping to hasty conclusions, and ignoring evidence that contradicts their hypotheses^37^), and (3) to experience hallucinations (via the Launay-Slade Hallucinations Scale^38^). In the first experiment, we sought to establish whether blocking in a causal learning paradigm (in either its additive or non-additive form) would be predicted by teleological thinking in a between-subjects experiment (in which half of the subjects completed a non-additive blocking task, while the other half completed an additive blocking task). In the next two experiments, we looked at which of the two types of causal learning would predict teleological thinking by employing a within-subjects design, in which subjects now completed *both* additive and non-additive tasks. We then investigated the relationships found in the behavioral data with a computational model, which allowed us a framework to explain the contributions of associations, propositional rules, and prediction errors in teleological thought.

**Table 2:** Trial types for the binary reasoning task

If binary (1/0) reasoning		If non-binary reasoning	
P(fever) = Low(Mangos) + Low(Bananas)	P(fever) = Low(Mangos) + High(Bananas)	P(fever) = Low(Mangos) + Low(Bananas)	P(fever) = Low(Mangos) + High(Bananas)
(0.1)^a^ (1) + (0.1)(0)	(0.1)(1) + (0.9)(0)	(0.1)(0.7) + (0.1)(0.3)	(0.1)(0.7) + (0.9)(0.3)
(a) 0.1	(b) 0.1^b^	(c) 0.1	(d) 0.34^c^

a Suppose we model “low” as 0.1, and “high” as 0.9.

b If people binarize probabilities, the difference between (a) and (b) should be 0.

c If people do not binarize probabilities, the difference between (a) and (b) should be above 0.

## Results

### Summary of blocking results across experiments

#### Results from Experiment 1

**Figure 2:**
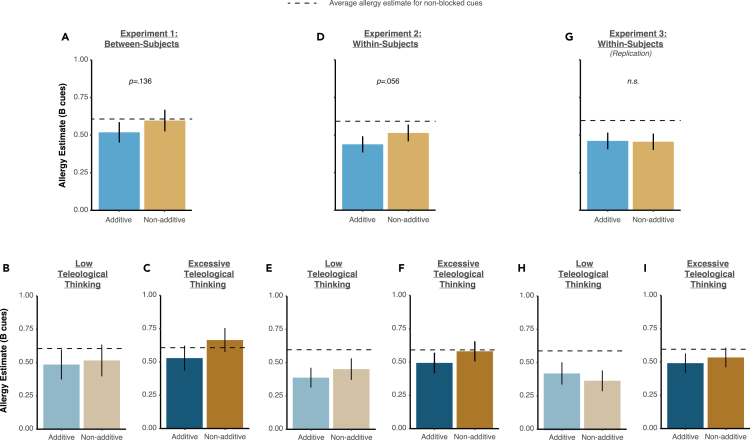
Blocking results across experiments (A, D, and G) Additive and non-additive blocking scores (reflected in allergy estimates for B cues). Blue bars represent additive blocking, yellow bars represent non-additive blocking. Horizontal gray dashed line represents average allergy estimates for non-B cues, as a baseline. Error bars reflect 95% confidence intervals. (B, C, E, F, H, and I) Additive and non-additive blocking scores, broken down by teleological thinking scores.

We verified the blocking effect across additive and non-additive blocking. These results are depicted in Figure 2A, in which the y axis depicts the mean “allergy” estimate for B cues (with a smaller value reflecting stronger blocking). For both additive and non-additive cases, we observed blocking (with both bars below the average allergy estimates in non-blocked cues), though blocking was greater in the additive than in the non-additive case. These results were confirmed with further statistical analyses. Subjects reported “no allergy” more for B cues (*M* = 0.55, *SD* = 0.38) than for D cues (*M* = 0.61, *SD* = 0.17; i.e., blocking), *t*(199) = 2.09, p = 0.037, *d* = 0.15, with the magnitude of blocking for B cues being marginally stronger in the additive than in the non-additive case, *M* = 0.51 vs. *M* = 0.59, *t*(198) = 1.50, p = 0.136, *d* = 0.21.

In additive blocking, subjects reported “no allergy” more for B cues (*M* = 0.51) than for D cues (*M* = 0.59), *t*(99) = 2.23, p = 0.028, *d* = 0.22. In non-additive blocking, there was no reliable blocking effect (B vs. D cues, *M* = 0.59 vs. *M* = 0.62, *t*(99) = 0.794, p = 0.429, *d* = 0.08). We then broke down subjects in the non-additive blocking condition by low and high BPRE (using the median BPRE score, *in lieu* of a clinical cutoff). Low teleological thinking subjects reported “no allergy” more for B cues (*M* = 0.50) than for D cues (*M* = 0.61), *t*(96) = 2.46, p = 0.016, *d* = 0.25, while high teleological thinking subjects did not show reliable blocking (B vs. D cues, *M* = 0.59 vs. *M* = 0.61, *t*(99) = 0.427, p = 0.670, *d* = 0.04).

#### Results from Experiment 2

We verified the blocking effect across additive and non-additive blocking. Subjects reported “no allergy” more for B cues (*M* = 0.48) than for D cues (*M* = 0.85), *t*(199) = 12.85, p < 0.001, *d* = 0.91. In additive blocking, subjects reported “no allergy” more for B cues (*M* = 0.44) than for D cues (*M* = 0.82), *t*(199) = 10.93, p < 0.001, *d* = 0.77. In non-additive blocking, subjects reported “no allergy” more for B cues (*M* = 0.51) than for D cues (*M* = 0.87), *t*(199) = 10.78, p < 0.001, *d* = 0.76. Next, we examined the predictive power of blocking for the variables of interest. For belief in the purpose of random events, non-additive blocking (*β* = 0.28, p = 0.039) predicted more variance over additive blocking (*β* = 0.21, p = 0.124). For binary reasoning, additive blocking (*β* = 7.61, p = 0.058) predicted more variance over non-additive blocking (*β* = −3.93, p = 0.307). Distress (measured from the PDI) was correlated with the paranoia (Spearman’s ρ = 0.57, p < 0.001), BPRE (ρ = 0.25, p < 0.001), hallucination tendencies (ρ = 0.47, p < 0.001), but not with binary reasoning (ρ = −0.03, p = 0.655).

#### Results from Experiment 3

We verified the blocking effect across additive and non-additive blocking. Subjects reported “no allergy” more for B cues (*M* = 0.46) than for D cues (*M* = 0.86), *t*(199) = 14.05, p < 0.001, *d* = 0.99. In additive blocking, subjects reported “no allergy” more for B cues (*M* = 0.46) than for D cues (*M* = 0.84), *t*(199) = 11.01, p < 0.001, *d* = 0.78. In non-additive blocking, subjects reported “no allergy” more for B cues (*M* = 0.46) than for D cues (*M* = 0.87), *t*(199) = 12.73, p < 0.001, *d* = 0.90. Next, we examined the predictive power of blocking for the variables of interest. For belief in the purpose of random events, non-additive blocking (*β* = 0.28, p = 0.035) predicted more variance over additive blocking (*β* = 0.03, p = 0.809). For binary reasoning, additive blocking (*β* = 5.78, p = 0.132) predicted more variance over non-additive blocking (*β* = −4.36, p = 0.260). Distress (measured from the PDI) was correlated with the paranoia (Spearman’s ρ = 0.57, p < 0.001), BPRE (ρ = 0.25, p < 0.001), hallucination tendencies (ρ = 0.47, p < 0.001), but not with binary reasoning (ρ = −0.03, p = 0.655).

### Associations vs. reasoning (between-subjects)

**Figure 3:**
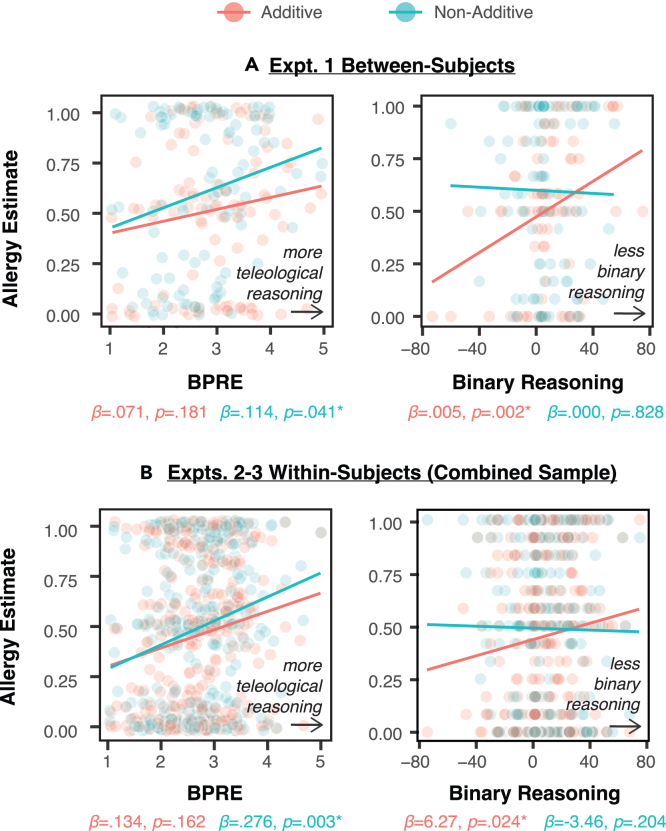
Double dissociation between non-additive and additive blocking (A) Scatterplots for the between-subjects experiment (Experiment 1). (B) Scatterplots for the within-subjects experiment (Experiments 2–3 combined).

Next, we examined the correlates of blocking in each group. The relationship between blocking and teleological thinking can be appreciated visually in Figures 2B and 2C. In particular, blocking was intact in the low teleological thinking group (via median split; Figure 2B), but not in the high teleological thinking group (Figure 2C), with the main effect being driven by non-additive blocking. These initial figure impressions were confirmed with further statistical tests. For non-additive blocking, only the Belief in the Purpose of Random Events (BPRE) predicted unique variance in the magnitude of blocking (*β* = 0.11, p = 0.041). For additive blocking, only the tendency to binarize beliefs predicted the magnitude of blocking (*β* = 0.01, p = 0.002). These key relationships are depicted in Figure 3A, and all beta-weights and p values are presented in Table 3. Distress from delusion-like beliefs (measured from the PDI, higher in patients with schizophrenia and previously related to neural prediction error responses; Corlett & Fletcher, 2012^33^) was correlated with the paranoia (Spearman’s ρ = 0.71, p < 0.001), BPRE (ρ = 0.52, p < 0.001), and hallucination tendencies (ρ = 0.23, p = 0.001), but not with binary reasoning (ρ = −0.07, p = 0.306). It appears that teleological thinking is a fundamental component of delusion-like thinking and that it is subserved by associative rather than propositional mechanisms. Furthermore, ratifying our approach, additive blocking (which we align with reasoning processes) was uniquely explained by participants’ bias toward binary reasoning (but not their excess teleology, delusions, or hallucinations).

**Table 3:** Regression weights and p values (with *survey scores* as predictors)

Predictors \ Response Variables	Non-additive	Additive
Distress from delusions	−0.005 (p = 0.959)	−0.081 (p = 0.413)
Paranoia	−0.002 (p = 0.788)	0.006 (p = 0.321)
Belief in the purpose of random events	0.114 (p = 0.041)∗	0.071 (p = 0.181)
Hallucination tendencies	−0.024 (p = 0.502)	−0.005 (p = 0.892)
Binary reasoning	0.000 (p = 0.828)	0.005 (p = 0.002)∗

### Associations vs. reasoning (within-subjects)

To more directly compare the predictive power of additive versus non-additive blocking, we conducted the next experiment (and its pre-registered replication) as a within-subjects paradigm. Results are depicted in Figures 2D–2I, but we report the combined results of both experiments here. Subjects again reported “no allergy” more for B cues (*M* = 0.47, *SD* = 0.34) than for D cues (*M* = 0.85, *SD* = 0.19; i.e., blocking), *t*(399) = 19.02, p < 0.001, *d* = 0.95, with the magnitude of blocking for B cues being marginally stronger in the additive than in the non-additive case (E2: *M* = 0.44 vs. *M* = 0.51, *t*(398) = 1.92, p = 0.056, *d* = 0.19; E3: *M* = 0.46 vs. *M* = 0.46, *t*(398) = 0.09, p = 0.924, *d* = 0.009).

Across all the regression models (one for each of the five survey measures), only two models showed significant results. These key relationships are depicted in Figure 3B, and all beta-weights and p values are presented in Table 4. For BPRE, non-additive blocking (*β* = 0.276, p = 0.003) explained unique variance when compared directly against additive blocking (*β* = 0.134, p = 0.162). For binary reasoning, additive blocking (*β* = 6.26, p = 0.023) explained unique variance when compared directly against non-additive blocking (*β* = −3.46, p = 0.204). To confirm the double dissociation, we fit two logistic mixed models. There was a significant interaction between BPRE and blocking type (*β* = 0.18, p < 0.05; i.e., difference in slopes between additive and non-additive blocking), and between binary reasoning and blocking type (*β* = −0.15, p < 0.001).

**Table 4:** Regression weights and p values (with *blocking types* as predictors)

Predictors \ Response Variables	Distress	Paranoia	BPRE	Hallucination tendencies	Binary reasoning
Non-additive	−0.016 (p = 0.822)	0.769 (p = 0.513)	0.276 (p = 0.003)∗	−0.078 (p = 0.551)	−3.46 (p = 0.204)
Additive	0.074 (p = 0.316)	0.488 (p = 0.681)	0.134 (p = 0.162)	0.174 (p = 0.190)	6.27 (p = 0.024)∗

**Figure 4:**
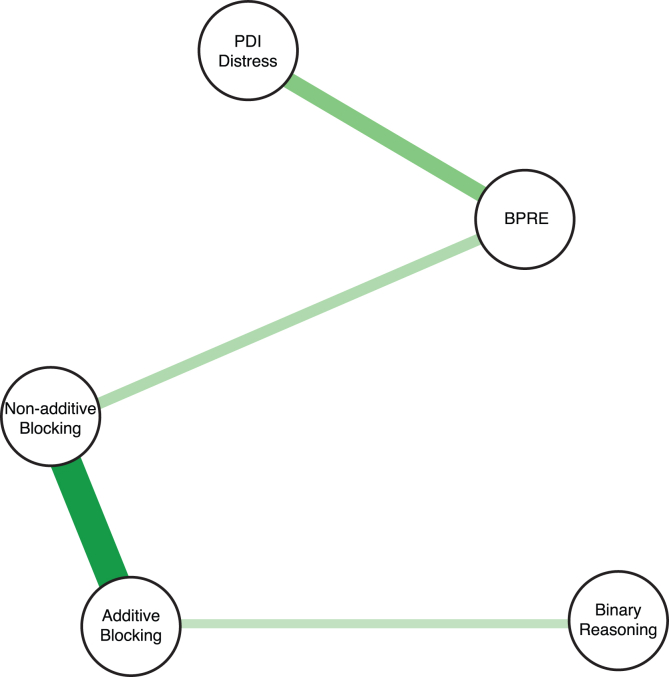
Bayesian Gaussian Graphical Model Each node (or vertex) represents one of the next values: non-additive blocking score; additive blocking score; binary reasoning; PDI-distress score. Each edge (or link) represents the estimate of a model predicting a node *i* with a node *j* adjusted by all the other nodes. The thickness of the node connections represents the strength of the association, and the color the direction (green is positive, red is negative).

Distress from delusion-like beliefs was correlated with the paranoia (Spearman’s ρ = 0.57, p < 0.001), BPRE (ρ = 0.20, p = 0.003), hallucination tendencies (ρ = 0.49, p < 0.001), but not with binary reasoning (ρ = 0.07, p = 0.319). These key relationships were confirmed in an even more conservative model (using Bayesian Gaussian Graphical Models [BGGM]^39^^,^^40^) — and a summary of the key relationships are depicted in Figure 4. Altogether, these analyses show the strong positive relation between both blocking types, and confirm the double dissociation between non-additive blocking and belief in the purpose of random events/additive blocking and binary reasoning. It also implies that belief in the purpose of random events may undergird the relationship between non-additive blocking and distress from delusions.

### Associations vs. reasoning (computational modeling)

In order to explore the computational bases of the relationship between teleology and association, we fit computational learning models to each participant’s binary predictive responses. The winning model (for details on model comparisons, see STAR Methods) had three free parameters: α (learning rate) which represents relevance to the current prediction error; β (inverse temperature) which represents the tradeoff between exploration/exploitation; τ (tau; sharpness of the noisy-max function^41^) which represents how equally associative strength or weights will be considered to produce the outcome expectation.

**Figure 5:**
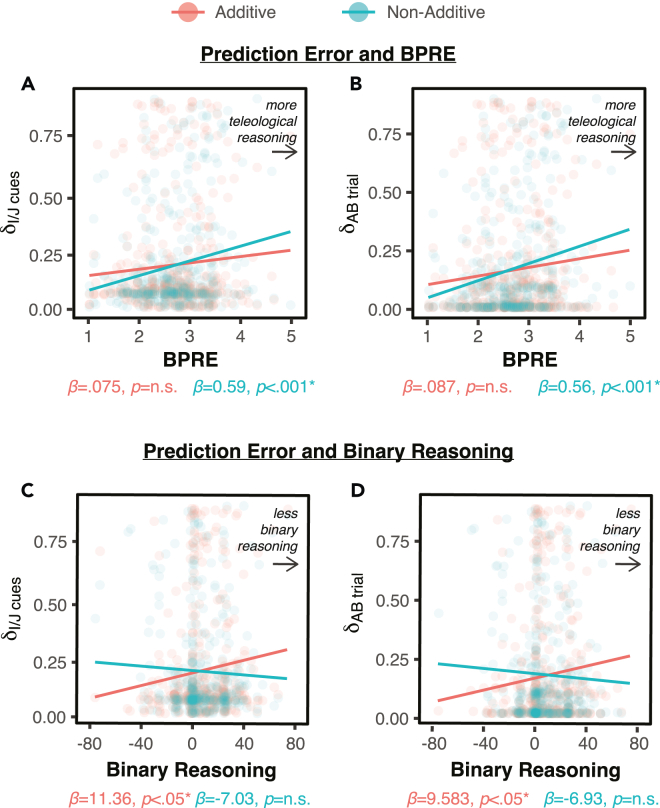
Prediction error, learning model, and questionnaires Computational models run on the within-subject data. (A and C) Prediction error (δ) average for all trial types during pre-learning per participant as a function of BPRE and binary reasoning, respectively. (B and D) δ average for AB trial type during blocking per participant as a function of BPRE and binary reasoning, respectively.

In order to evaluate aberrant prediction errors with the best model (Model 1, see STAR Methods), we simulated the task under the best fitted parameters for each participant and averaged the prediction error for the pre-training and blocking phases. We then fit linear models with the two key variables we have been repeatedly seeing associations with the two types of blocking: BPRE and binary reasoning (see Figure 5). On one hand, there was a positive relationship between prediction error and BPRE only for non-additive blocking, but not for additive blocking (prediction errors during pre-training: additive *β* = 0.075, *p* = n.s.; non-additive *β* = 0.59, p < 0.001; prediction errors during blocking: additive *β* = 0.087, *p* = n.s.; non-additive *β* = 0.56, p < 0.001) — and this interaction was significant (*β* = 0.03, p < 0.001). On the other hand, there was a positive relationship between prediction error and binary reasoning only for additive blocking, but not for non-additive blocking (prediction errors during pre-training: additive *β* = 11.363, p < 0.05; non-additive *β* = −7.03, *p* = n.s.; prediction errors during blocking: additive *β* = 9.583, p < 0.05; non-additive *β* = −6927, *p* = n.s.) — and this interaction was significant (*β* = −0.001, p < 0.001).

These computational modeling results demonstrate the double dissociation we had found in the behavioral data: teleological thinking tendencies are predicted by non-additive blocking (via prediction error processing), but not by additive blocking (which is only correlated with binary reasoning, and not teleological thinking).

## Discussion

Teleological thinking, in previous work, has been defined in terms of “beliefs”,^9^ “social cognitive biases”,^9^ and indeed carries “reasoning” in its very name (as it is used interchangeably with ‘purpose-based’ reasoning^1^^,^^42^^,^^43^) — which is why it might be surprising to learn of the relationship between teleological thinking and low-level associative learning, and *not* learning via propositional reasoning. The key result across experiments can be summarized as such: aberrant prediction errors augured weaker non-additive blocking, which predicted tendencies to engage in teleological thinking, which was consistently correlated with distress from delusional beliefs. This pattern of results was demonstrated in both behavioral and computational modeling data, and withstood even more conservative regression models, accounting for the variance explained by other variables. In other words, excessive teleological thinking (which correlated with distress from delusional beliefs) was not merely a function of weaker or stronger reasoning abilities, but rather was a function of tendencies to learn from and ascribe causal power to irrelevant cues.

This chain of relationships is consistent with previous work relating dopamine and prediction errors to causal learning in experimental animals,^16^ and relating weaker blocking with psychosis risk and amphetamine administration.^44^ But our data suggest that teleological thinking may broker the relationship between weaker blocking and psychosis. Preclinical data already tend to suggest that dopamine signaling is intimately related to prediction error processing, clinical data suggest aberrant dopamine dynamics contribute to delusional ideation,^45^^,^^46^ and contemporary dopamine modeling data suggest that, rather than reward prediction errors, dopamine may instantiate inferences about causal relationships.^47^ Taken together with the present results, we suggest that teleological thinking is underwritten by promiscuous dopaminergic prediction error signals which imbue irrelevant and merely contiguous stimuli and events with causal significance, culminating in delusion-like ideas.

This new finding is especially interesting in light of how teleological thinking has been described in previous work. In particular, tendencies to explain the world in terms of a larger purpose or design have been theorized to be rooted in our tendencies to perceive intention and agency in the world.^48^ If non-additive blocking predicts distress from delusional beliefs by way of teleological thinking, then this might suggest a sort of “atomism”, and explain why contents of delusions are social in nature, without needing to appeal to recursive tracking of reputations and coalitions.^49^^,^^50^ In particular, if causal associative learning is fundamental to teleology, inferred causes tend to imply agents, and, since agents are typically human, we arrive at the social contents of delusion-like ideas.^51^

The second pattern that was consistent throughout the experiments was that additive blocking did *not* explain unique variance in teleological thinking beyond what was already explained by non-additive blocking. Additive and non-additive blocking are highly correlated — which suggests that both higher-level reasoning over rules and low-level associations between cues and outcomes probably co-exist and contribute to causal learning. But these two types of blocking are nonetheless distinct, as prediction errors correlated in different ways with the relevant variables (BPRE and binary reasoning). This implies that non-associative knowledge mechanisms must be invoked to explain blocking under additivity assumptions.^52^ They may be separate systems, running in parallel.^53^ This may explain the seeming paradox in which scientific understanding and teleological thinking can be found in the same person.^11^ For instance, it has been argued that cognition favors Type-I errors instead of Type-II ones (i.e., overpredicting or having a causal illusion), such that it is better to overpredict a predator in the absence of a clear cause, and it may be better to feel optimistic even if there is no real evidence for this.^55^ The first example may be mediated by associative learning, and the second by reasoning processes. Or teleology and knowledge structures can co-occur, but sensitivity to propositional knowledge does not necessarily undermine teleology. These results could have implications for how we think about other illusions of causality in everyday thinking.^54^

One of the key contributions of these studies is the direct comparison between association-based versus rule-based processing in causal learning and teleological thinking. Often, evidence for associations versus propositions live in independent literatures — whereas here we argue that finding ways to test these two pathways using the same experimental paradigm can yield new insights that would not have otherwise been found (i.e., that association-based vs. rule-based learning are actually correlated, but only one has predictive power for the phenomenon of interest). This raises new questions about the nature of the underlying representations and computations themselves. For instance, one way that additivity assumptions might infiltrate an associative model is through stimulus representation. In our winning model, cues are combined in terms of their relative predictions of outcome. In contrast, if the model allowed for “compositionality” — where cues are combined into a single configural entity during blocking trials, and prediction errors operate over that configural cue, then associative models can evince more blocking under additivity.^56^ Note that this is different from a change in prediction error or its weighting — and future work can explore the explanatory power of such a model.

Throughout this study, we have emphasized two new aspects about teleological thinking — its intimate relationship with associative mechanisms of causal learning, and the fact that only it (and not binary reasoning) correlates with delusion-like beliefs. While our primary measures involved connecting teleological thinking with the *distress* experienced from the latter, we want to end by noting that teleological thinking must cut both ways. Ultimately, the tendencies to ascribe purpose and intent to events that we experience may be distressing for some (in the case of paranoia), but comforting for others (in the case of pronoia). Here we suggest that how these teleological explanations play out may rest not just on how we think or reason about purpose or intent — but on more basic mechanisms through which we establish connections and causes in the world.

### Limitations of the study

The effects reported here were replicated across between- and within-subject designs, and across multiple online platforms (Mturk and Prolific), and across multiple different regression models. However, there remain several open questions about the nature and scope of the effects reported here. First, while non-additive blocking could be explained through prediction errors in the computational models, future work can explore the more propositional mechanisms that could support the additive version of blocking. Second, future work can draw out and test further the implications of the current results. In particular, if teleological thinking is ultimately supported by a mere *associative* (and thus essentially correlational) mechanism, then this might not fully capture *causation* in the strictest sense. It is possible that to capture causality in the strict sense, other processes may have to be recruited (e.g., counterfactual thinking). Third, the current study only tested one variant of teleological thinking, via the validated Belief in the Purpose of Random Events survey, so it remains unknown whether or how teleological thinking in other forms (e.g., reasoning about the purpose of objects) may be impacted by the processes described here.

## STAR★Methods

### Key resources table

**Table:** 

REAGENT or RESOURCE	SOURCE	IDENTIFIER
**Deposited data**		

Raw and analyzed data	This paper	Data S1

**Software and algorithms**		

Custom experimental code	This paper	https://osf.io/w974g/?view_only=25ebc8ee3f594e7583ed5c8ff2ae45a4

### Resource availability

#### Lead contact

Further information and requests for resources should be directed to and will be fulfilled by the lead contact, Philip Corlett (philip.corlett@yale.edu).

#### Materials availability

This study did not generate any new unique reagents.

### Experiment model and subject participant details

For Experiments 1–3, 200 unique subjects (E1 age and gender information were not collected; E2 mean age: 35.0, 46.2% female, 50.4% male, 3.4% other; E3 mean age: 35.7, 51.2% female, 46.7% male, 2.0% other), recruited using the Amazon Mechanical Turk (mTurk) online platform (Experiment 1) and the Prolific platform (Experiments 2–3), participated for monetary compensation. mTurk prescreening criteria required subjects to be in the United States, to have an mTurk task approval rate of at least 90%, to have had previously completed at least 100 mTurk tasks, to have no prior participation in another experiment from this project, and to use a laptop or desktop computer (but not a phone or tablet). Prolific prescreening criteria required subjects to be in the United States, to have no prior participation in another experiment from this project, and to use a laptop or desktop computer. The sample size was chosen before data collection began, was pre-registered, and was fixed to be identical for Experiments 1–3. All experimental methods and procedures were approved by the Yale University Institutional Review Board, and all subjects read and completed a consent form outlining their risks, benefits, compensation, and confidentiality before participating in the experiment. The preregistered methods and analyses can be viewed at: https://aspredicted.org/KTJ_6TM (Experiment 3).

### Method details

#### Experiment 1: Associations vs. reasoning? (Between-subjects)

After agreeing to participate, subjects were redirected to a website where stimulus presentation and data collection were controlled via custom software written using a combination of HTML, CSS, JavaScript, PHP, and JsPsych libraries.^56^ Subjects completed the experiment in fullscreen mode on either a laptop or desktop computer. (Since the experiment was rendered on subjects’ own web browsers, viewing distance, screen size, and display resolutions could vary dramatically, so we report stimulus dimensions below using pixel [px] values.)

At the beginning of the experiment, subjects were first told the following cover story: “You will be asked to imagine that you are an allergist (someone who tries to discover the cause of allergic reactions in people). You have been presented with a new patient who suffers from allergic reactions following some types of food, but not others. You arrange for skin allergy prick tests for different types of foods, and observe the magnitude of the allergy reaction.” For each trial, subjects were then simply asked to make a prediction about the allergic reaction that the patient would experience given a food cue. First, they saw a fixation cross at the center of the display for 500ms, followed by a food cue (400px x 400px) at the center of the display. Right below the food cue was a prompt that said “Input your prediction”, and a 250px x 250px feedback box below this. Subjects could press one of four keys to indicate their prediction, with each key corresponding to (1) no reaction (−), (2) small allergic reaction (+), (3) medium allergic reaction (++), and (4) strong allergic reaction (+++). Depending on the key press, an image of the predicted allergic reaction would appear in the feedback box. Subjects were given 3s to respond, after which the trial automatically proceeded. When subjects were given feedback, they were presented an image of the actual allergic reaction in the feedback box. The experiment proceeded to the next trial.

A subject could be randomly assigned to one of two types of tasks: (1) Non-additive blocking, or (2) Additive blocking.

##### *Non-additive blocking design*

Each subject went through four phases: (1) Pre-Learning, (2) Learning, (3) Blocking, (4) Test. In the Pre-Learning and Learning phases, subjects first learned which food cues cause an allergic reaction (e.g., that food cue A caused an allergic reaction of the strength +, or that food cue C does not cause an allergic reaction). In the Blocking phase, subjects sometimes encountered the allergic food cues paired with a novel food cue, and that the pair of cues causes the same magnitude of an allergic reaction (e.g., that food cue A paired with food cue B causes an allergic reaction of the strength +) — and other times, they encountered the non-allergic food cues with a novel food cue, and that the pair of cues causes an allergic reaction (e.g., that food cause C paired with food cue D causes an allergic reaction). To balance responses (of allergy versus no-allergy), the Blocking phase also included “no-allergy” trials, in which a food cue was presented alone (food cue E), and did not cause an allergic reaction. In this phase, subjects should learn that the novel food cue B does not cause an allergic reaction (i.e., “blocking”), and that the novel food cue C does cause an allergic reaction. In the Test phase, subjects were presented with B, D, and E food cues. No feedback was given in the Test phase. Trial types are depicted in Table 1 in the main text. Subjects saw 6 repetitions of each trial type. Food cues were adapted from previous work,^33^ and which food cues were assigned to which trial types were randomly determined per subject.

##### *Additive blocking design*

This design is identical to the Non-additive blocking design, except where noted. In the Pre-Learning phase, subjects this time learned a “causal additivity” rule: that a food cue (e.g., food cue I) could cause one magnitude of an allergic reaction (of a strength +), that a different food cue (e.g., food cue J) could cause a different magnitude of an allergic reaction (of a strength ++), and that both of these combined could cause a strong allergic reaction (of a strength +++).

##### *Surveys*

At the end of the experiment, subjects were redirected to a Qualtrics compilation of off-the-shelf surveys measuring different symptoms of psychosis (e.g., delusional thinking, paranoia, hallucinations, teleological reasoning, and probabilistic reasoning). Subjects completed the following surveys (with the order randomly determined per subject).

(a) *Peters Delusion Inventory* (PDI^34^). Subjects completed a 21-item questionnaire. Items were adapted for healthy, nonpsychotic individuals by prefacing items with a relative, “as if” extension (e.g., “Does it ever feel as if … ?”). For each item, subjects first reported whether they endorsed the relevant belief (e.g., “Do you ever feel as if things in magazines or on TV were written especially for you?”, or “Have your thoughts ever been so vivid that you were worried other people would hear them?”), and if they did, they then filled out three 5-point Likert scales assessing the degree of distress, pre-occupation, and conviction associated with the belief.
(b) *Revised Green* et al. *Paranoid Thought Scale* (R-GPTS^35^). Subjects completed two sections (R-GPTS A [for self-reference delusions], and B [for paranoid delusions]), where they simply rated the degree to which they thought or felt a statement (e.g., “I spent time thinking about friends gossiping about me”, or “People have been hostile toward me on purpose”) in the last month — on the scale of 0 (Not at all) to 4 (Totally).
(c) *Belief in the Purpose of Random Events* (BPRE^2^). Subjects were asked to picture a situation (e.g., “A person that you are attracted to kisses you in the middle of the street”) for 5 s — and were then given a scenario (e.g., “You start going out together”). Subjects were then asked to rate the extent to which they believed that the first situation happened for a purpose — on the scale of 1 (The event definitely did not have a purpose) to 5 (The event clearly had a purpose).
(d) *Launay-Slade Hallucination Scale - Revised* (LSHS-R^38^). Subjects rated the degree to which a statement applied to them (e.g., “In my daydreams, I can hear the sound of a tune almost as clearly as if I were actually listening to it”, or “I often hear a voice speaking my thoughts aloud”) — on the scale of 1 (Certainly applies to me) to 5 (Certainly does not apply to me).
(e) *Binary Beliefs Task*.^36^ Subjects were presented different probabilities (e.g., mushrooms cause rashes, bananas cause vomiting, and that mangos cause both rashes and vomiting), and are asked to estimate the probability that a person ate something, given the following symptoms (e.g., “A patient shows both rashes and vomiting. How likely is it that they had bananas?”). In this case, bananas should have a low probability, and mangos (as the simpler explanation) should have a high probability. The critical question, however, is how subjects then use these probabilities to reason about a different outcome. So now, suppose these different foods can potentially cause a fever, and we provide the probabilities of fever given a particular food (e.g., a low probability — “When a patient eats mangos, they rarely have a fever.” versus a high probability — “When a patient eats bananas, they usually have a fever”). In this case, typically, the probability of an outcome is defined as: P(fever) = P(fever|bananas) x P(bananas) + P(fever|mangos) x P(mangos), but previous work has found that instead of being sensitive to the actual probabilities, people instead just binarize — treating P(bananas) as 0, and P(mangos) as 1. We computed a “binary reasoning” score by obtaining two different types of responses from the subjects (depicted in the two columns of Low-Low vs. Low-High in Table 2 in the main text). People who binarize probabilities should have a score closer to 0 (columns 1 and 2 in Table 2 in the main text), and people who do not binarize should have a score above 0 (columns 3 and 4 in Table 2 in the main text).

#### Experiments 2–3: Associations vs. reasoning? (Within-subjects)

These experiments were identical to Experiment 1 except where noted. In order to see which of the two types of blocking (additive versus non-additive) predicted specific correlates more than the other, we conducted a within-subjects version of Experiment 1: subjects completed *both* tasks (with the order randomly determined per subject), and with the surveys completed in between the two tasks.

### Quantification and statistical analysis

All data across all the experiments here can be found in OSF: https://osf.io/w974g/?view_only=25ebc8ee3f594e7583ed5c8ff2ae45a4.

#### *Subject exclusions*

Per the preregistered exclusion criteria, subjects in Experiment 1 were excluded from the analyses (with replacement) for the following criteria: (1) subjects whose total completion time was more than 2 standard deviations from the grand population mean (n = 9), and (2) subjects whose accuracy for no-allergy “catch” trials was 50% or below (n = 23); in Experiment 2: (1) subjects whose total completion time was more than 2 standard deviations from the grand population mean (n = 9), and (2) subjects whose accuracy for no-allergy “catch” trials was 50% or below (n = 19); in Experiment 3: (1) subjects whose total completion time was more than 2 standard deviations from the grand population mean (n = 12), and (2) subjects whose accuracy for no-allergy “catch” trials was 50% or below (n = 24).

#### *General analysis*

To verify that blocking occurred in our subjects, we coded (−) responses as 0 for “no allergy”, and (+/++/+++) responses as 1 for “allergy”. As a measure of blocking, we will use a within-subjects t-test to compare the average allergic reaction response of subjects for B cues (the blocked stimulus) versus D cues (the non-blocked stimulus). In Experiment 1, to see whether different correlates explain different types of blocking, we set-up two regression models for the two types of blocking, with the following five predictors (distress from delusions [PDI-distress], paranoia [RGPTS-B], belief in purpose of random events [BPRE], hallucination tendencies [LSHS-R], binary reasoning [binary beliefs task]). In Experiments 2–3, to determine the predictive strength of additive versus non-additive blocking in terms of explaining different symptoms of psychosis, we set-up five regression models, one for each of the five survey measures (distress from delusions [PDI-distress], paranoia [RGPTS-B], belief in the purpose of random events [BPRE], hallucination tendencies [LSHS-R], binary reasoning [binary beliefs task]), with the two types of blocking as predictors (additive versus non-additive blocking). Across all experiments, to see how different measures relate to psychosis, we will correlate RGPTS-B, BPRE, LSHS, and the binary beliefs task with the distress scores from the PDI.

#### *Computational modeling*

We modeled the predictive response of the Kamin blocking task with five models. We compared models with Bayesian Information Criterion (BIC), which punished a model for complexity, and with their hit rates, average of correct predicted responses. First, we present the winning model (Model 1). We used the winning model’s fitted parameters to simulate the underlying learning values (e.g., $\delta_{t}$) for each participant.

The models had two rules: *i)* decision (how predictive responses are made), and *ii)* learning (how the weights or associative strength, $\vec{v_{t}}$, are updated). The decision rule defines the probability of a predictive responses and was model with a softmax function (σ; sigma):

(Equation 1) $${p\left(response\right)}_{t}=\sigma \left(x_{t},\beta \right)=\frac{1}{\left(1+\exp\left(-\beta *x_{t}\right)\right)}\text{;}$$

where $\beta \in R^{+}$ (beta) and its corresponding steepness or inverse temperature parameter. To produce responses, the softmax input x was given by:

(Equation 2) $$x_{t}=\vec{i_{t}}\cdot \vec{v_{t}}\text{.}$$

where $\vec{i_{t}}$ is a boolean vector representing presence/absence of cues, and $\vec{v_{t}}$ is vector with the associative strength or weights for each cue.

##### Winning model

Model 1 or model *“noisy-MAX”*,^41^^,^^57^ has the fundamental characteristic where the cue/input with larger $v_{t}$ is the one with a larger contribution to the overall outcome expectation ($\xi_{t}$, xi). For this model $\xi_{t}=noisyMax\left(\vec{w_{t}},\tau \right)$, where $\vec{w_{t}}$ is a vector with the weights, $v_{t}$, of only the present cues (i.e., $\vec{i_{t}}=1$). The form of the noisy-MAX function is:

(Equation 3) $$\xi_{t}=sum\;\left[\vec{w_{t}}\left(\frac{e^{\frac{\vec{w_{t}}}{\tau }}}{sum\left|\left(e^{\frac{\vec{w_{t}}}{\tau }}\right)\right|}\right)\right];$$

where $\tau \in R^{+}$ (tau) modulates the proportion of each w that is considered for the overall sum. Thus when τ→∞, each w contributes equally, and when τ→0 the sum considers only the largest w. The next part of the model takes the classical form of the Rescorla-Wagner model.^15^ Where, the prediction error (δ; delta) is the difference between the outcome ($o_{t}$) and $\xi_{t}$:

(Equation 4) $$\delta_{t}=o_{t}-\xi_{t}\text{;}$$

then the next $\vec{v_{t+1}}$ was updated for every trial by adding the $\delta_{t}$ weighted by a learning rate (α; alpha) to the current $\vec{v_{t}}$, in the form:

(Equation 5) $$\vec{v_{t+1}}=\vec{v_{t}}+\alpha \vec{i_{t}}\delta_{t}\text{;}$$

where $\alpha \in R^{+};\;\alpha <1$. The initial values of all the cues’ associative strength $\vec{v_{t}}$ were 0. The outcome was encoded as +1 and −1. As a model sanity check, we used the fitted parameters from all the participants to model the responses. Then we recover the fitted parameters from the simulated responses. All the correlations between fitted and recovery parameters were positive and significant.

##### Alternatives models

In these sections we describe the four comparing models. Model 2, overexpectation, allows the possibility that the outcome prediction is higher than the outcome. Model 3, bounding outcome prediction, does not allow overexpectation because the outcome prediction is bounding between 0 and 1; Model 4, configural model, where the compound are represented independently of the individual elements, i.e., as a new input unit; and Model 5, Pearce-Hall model, similar to the Model 2 but with a volatile learning rate for each cue. The first four models are inspired by Rescorla-Wagner,^15^ and the fifth by Pearce-Hall learning model, i.e., with a volatile learning rate.^59^ All models’ free parameters α, β, τ, and η (see Model 5) were fitted to each task and each participant.

#### *Model comparison*

We fit five models to each task (see details below). In Experiment 1 each model to each participant, and Experiment 2 and 3 each model twice to each participant, one for each task: additive and non-additive. Figure S1 illustrates the models’ comparison in both BIC (low better) and hit rate (high better). The lowest BIC was obtained in Model 1, thus the best model. However, Model 3 seems to perform well, then we compare hit rates between both models. Given that hit rates do not distribute normally we employed Wilcoxon rank-sum test for Experiment 1 and Wilcoxon signed rank test for Experiments 2 and 3. In summary, the hit rates for Model 1 were significantly larger than Model 3, for Experiment 1 (Additive: W = 5892.5, p < 0.05; Non-additive: W = 5850.5, p < 0.05), Experiment 2 (Additive: W = 13656, p < 0.001; Non-additive: W = 11301, p < 0.001), and Experiment 3 (Additive: W = 14555, p < 0.001; Non-additive: W = 13347, p < 0.001). Thus Model 1 outperformed Model 3.

##### *Model 2 “Overexpectation Model”*

The input for σ, $x_{t}$, was a function of the associative strength or weights ($\vec{v_{t}}$) and the number of inputs (i.e., $sum\left(\vec{i_{t}}\right)$):

(Equation 6) $$x_{t}=\frac{\left[\left(\vec{v_{t}}-\left(\vec{1}-\vec{v_{t}}\right)\right)\cdot \vec{i_{t}}\right]}{sum\left(\vec{i_{t}}\right)}\text{,}$$

where $\vec{1}$ is a vector of 1’s from the same size as $\vec{v_{t}}$. In contrast to the previous model, this model $\xi_{t}$ was calculated as the dot product between the inputs and the weights (associative strength):

(Equation 7) $$\xi_{t}=\vec{i_{t}}\cdot \vec{v_{t}}\text{.}$$

The prediction error and the updating rule were the same as Model 1 (Equations 4 and 5). In order to allow overexpectation and σ(0,β)=0.5, the initial values of all the cues’ associative strength $\vec{v_{t}}$ were 0.5. The outcome was encoded as +1 and 0.

##### *Model 3 “Bounding Outcome Prediction Model”*

Used a boundary for $\xi_{t}$, thus the outcome $o_{t}$ is never overexpected. Thus:

(Equation 8) $$\xi_{t}={\sigma \left(\vec{i_{t}}\cdot \vec{v_{t}},1\right)}_{t}\text{;}$$

which implies that the maximum $\xi_{t}$ will never reach 1^60^ and this remove the possibility of over expectation (i.e., $\xi_{t}>1$), i.e., negative prediction errors in the presence of the outcome. Also, when $\vec{i_{t}}\cdot \vec{v_{t}}=0$ then $\xi_{t}$ will be 0.5. Thus, the starting values of $\vec{v_{t}}$ were 0. The outcome was encoded as +1 and 0.

##### *Model 4 “Configural Model”*

Same as Model 2 but the input pattern was changed. Here, each trial type was represented by a different input unit. The assumption of this model was that people encode compounds as a totally different ‘thing’. $\vec{v_{1}}=0.5$, and the outcome was encoded as +1 and 0.

##### *Model 5 “Pearce & Hall Model”*

This model resembles Model 2 but uses a dynamic learning rate ($\alpha_{t}$)^61^, which changed for very current cue at every trial as follows:

(Equation 9) $$\alpha_{t}=\eta \left|\delta_{t}\right|+\left(1-\eta \right)\alpha_{t-1}\text{;}$$

where $\eta \in R^{+};\;\eta <1$ and is the moving average weight to smoothening the $\alpha_{t}$ change. Thus:

(Equation 10) $$\vec{v_{t+1}}=\vec{v_{t}}+{\vec{\alpha }}_{t}⊗\vec{i_{t}}\delta_{t}\text{;}$$

where ⊗ is the hadamard product (element wise multiplication). Finally, $\vec{v_{1}}=0.5$, and the outcome was encoded as +1 and 0.

## Data Availability

• De-identified human data for all the experiments reported here are accessible in the Supplementary Data Archive, and in the study’s main repository: https://osf.io/w974g/?view_only=25ebc8ee3f594e7583ed5c8ff2ae45a4. • All original code is accessible in: https://osf.io/w974g/?view_only=25ebc8ee3f594e7583ed5c8ff2ae45a4. • Any additional information required to analyze the data reported in this paper is available from the lead contact upon request.

## Supplemental information

Document S1. Figure S1

Data S1. The raw data for each reported experiment
