# Supplementary material for: Excessive teleological thinking is driven by aberrant associations and not by failure of reasoning
Source: iScience. 2023 Aug 15;26(9):107643. doi: 10.1016/j.isci.2023.107643 (PMC10495659; doi:10.1016/j.isci.2023.107643)
Supplement: Document S1. Figure S1 [file mmc1.pdf]

iScience, Volume *26*

Supplemental information

Excessive teleological thinking is driven by aberrant associations and not by failure of reasoning

Joan Danielle K. Ongchoco, Santiago Castiello, and Philip R. Corlett

Excessive teleological thinking is driven by aberrant associations, not by failure of reasoning

Joan Danielle K. Ongchoco, Santiago Castiello, & Philip R. Corlett*

Yale University

Supplemental Figure

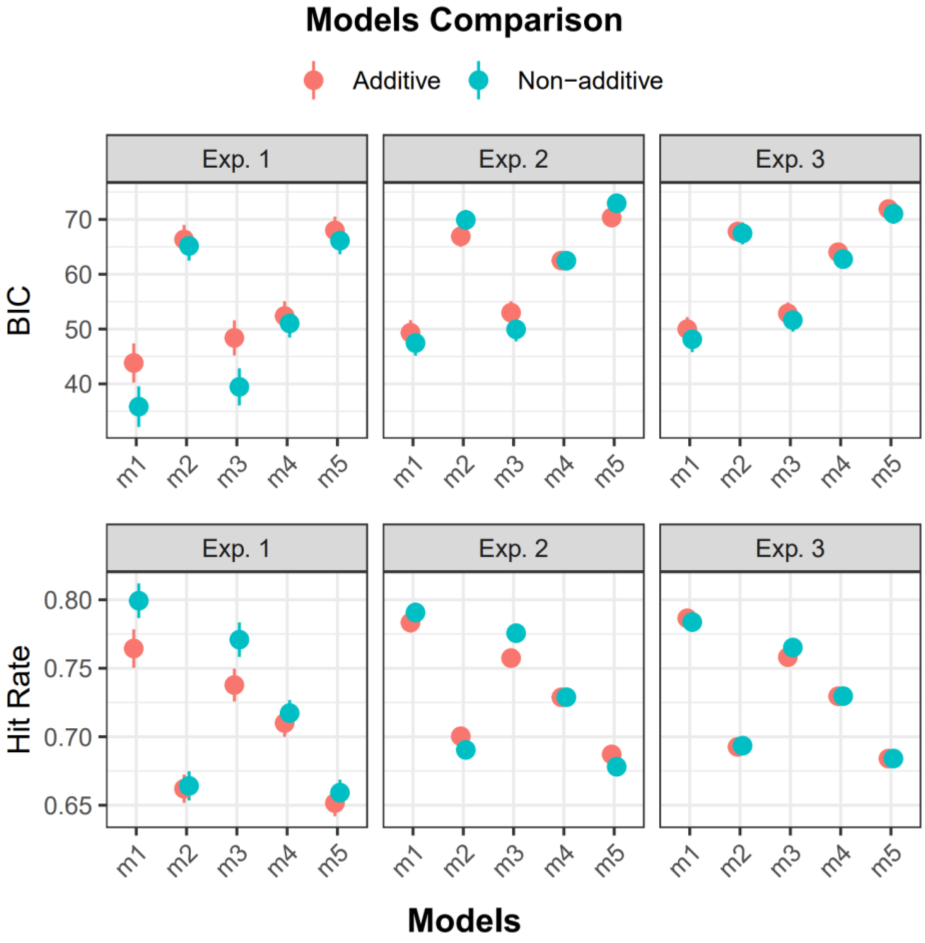

Figure S1. Model Comparison by Bayesian Information Criterion (BIC; lower better) and Hit Rate (higher better), **refers to Figure 5**. Model 1 "noise-MAX" differential contribution of weights to create the overall expectation. Model 2 "overexpectation", allows that outcome prediction is higher than the outcome. Model 3 "bounding outcome prediction", does not allow overexpectation. Model 4 "configural model", where the compound is represented independently of its individual elements. Model 5 "Pearce-Hall model", as model 2 but with a dynamic learning rate for each cue/input.
